# Superhalogen Anions Supported by the Systems Comprising Alternately Aligned Boron and Nitrogen Central Atoms

**DOI:** 10.3389/fchem.2022.863408

**Published:** 2022-04-21

**Authors:** Adrianna Cyraniak, Dawid Faron, Sylwia Freza, Iwona Anusiewicz, Piotr Skurski

**Affiliations:** Laboratory of Quantum Chemistry, Faculty of Chemistry, University of Gdańsk, Gdańsk, Poland

**Keywords:** polynuclear superhalogens, anions, electronic transmutation, excess electron, electron binding energies

## Abstract

Using DFT/(B3LYP/wB97XD/B2PLYPD) and OVGF electronic structure methods with flexible atomic orbital basis sets, we examined the series of polynuclear superhalogen anions matching the (BF_3_(BN)_*n*_F_4*n*+1_)^–^ formula (for *n* = 1-10,13,18-20) containing alternately aligned boron and nitrogen central atoms decorated with fluorine ligands. It was found that the equilibrium structures of these anions correspond to fully extended chains (with each B and N central atom surrounded by four substituents arranged in a tetrahedral manner) and thus mimic the globally stable fully extended (all-*trans*) conformations of higher n-alkanes. The vertical electron detachment energies of the (BF_3_(BN)_*n*_F_4*n*+1_)^–^ anions were found to exceed 8 eV in all cases and gradually increase with the increasing number of *n*. The approximate limiting value of vertical electron binding energy that could be achieved for such polynuclear superhalogen anions was estimated as equal to ca. 10.7 eV.

## Introduction

Superhalogens are commonly defined as the systems exhibiting the electron affinity (EA) larger than that of a chlorine atom (3.62 eV) (Hotop and Lineberger, 1985). The existence of such molecules and their corresponding anions (so-called superhalogen anions) was proposed in 1981 by Gutsev and Boldyrev who characterized (on the basis of theoretical calculations) several negatively charged compounds matching the (MF_k+1_)^–^ formula and a few (MO_(k+1)/2_)^–^ anions (where M is main-group central atom of maximal valence k) and confirmed their large excess electron binding energies (Gutsev and Boldyrev, 1981a). In the course of later studies, Gutsev and Boldyrev modified their formula describing superhalogens to cover various halogen atoms X that may serve as ligands in such systems (i.e., MX_k+1_ for the neutral molecules and (MX_k+1_)^–^ for the corresponding anions) (Gutsev and Boldyrev, 1981a; 1981b, 1984, 1985). On the other hand, many experimental attempts to measure the electronic stability of such anions were made, however, all determinations performed before 1999 were related to condensed phases only whereas the gas phase electron detachment energies had not been measured (Heni and Illenberger, 1985; Metz et al., 1988; Weaver et al., 1988; Compton, 1995; Huey et al., 1996; Wu et al., 1996; Taylor et al., 1998). The existence of superhalogen anions in gas phase was experimentally confirmed in 1999 by Wang and co-workers who measured gas-phase electron detachment energies of (MX_2_)^–^ (M = Li, Na; X = Cl, Br, I) systems (Wang et al., 1999). The measurements performed by the Wang group were supported by the advanced ab initio calculations executed by Boldyrev and Simons and it turned out that the vertical electron detachment energies (VDE) elucidated from the photoelectron spectra (i.e., 5.92 ± 0.04 (LiCl_2_)^–^, 5.42 ± 0.03 (LiBr_2_)^–^, 4.88 ± 0.03 (LiI_2_)^–^, 5.86 ± 0.06 (NaCl_2_)^–^, 5.36 ± 0.06 (NaBr_2_)^–^, and 4.84 ± 0.06 eV (NaI_2_)^–^) were in excellent agreement with the values predicted by theoretical calculations (Wang et al., 1999). This joined experimental and theoretical study of selected superhalogen anions was in fact a milestone achievement which both confirmed the existence and stability of such species in gas phase and demonstrated the usefulness of certain ab initio methods to predict their structures and excess electron binding energies. Since then, many research groups turned their attention to superhalogens which resulted in proposing numerous new compounds of that type in the following years. These studies included various alternative superhalogen anions utilizing non-metal or metalloid central atoms (e.g., (SiF_5_)^–^ (VDE = 9.32 eV), (GeF_5_)^–^ (VDE = 9.74 eV), (PF_6_)^–^ (VDE = 9.43 eV)) (Sobczyk et al., 2003; Marchaj et al., 2012), as well as numerous non-halogen ligands (such as halogenoids (Smuczyńska and Skurski, 2009; Li and Yin, 2021), electrophilic groups(Anusiewicz, 2009a), acidic functional groups (Anusiewicz, 2009b), and other halogen-free fragments (Sun et al., 2016c). In addition, it was found that even superhalogens themselves may act as effective ligands in superhalogen systems (Wang et al., 2009; Götz et al., 2010; Koirala et al., 2010, 2013; Willis et al., 2010; Feng et al., 2011; Paduani and Jena, 2012, 2013; Hou et al., 2013; Li et al., 2013; Tian et al., 2014; Yang et al., 2014; Sun W.-M. et al., 2016; Paduani, 2016; Liu et al., 2017). These and other superhalogen anions have recently been described in a comprehensive review article (Skurski, 2021).

Compounds exhibiting large excess electron binding energy or small ionization potential are of special interest because a wide range of new materials (such as organic superconductors, organic metals, ionic liquids, etc.) could be designed and synthesized on their base (Awasthi et al., 2021; Pandey, 2021). Since superhalogens represent the species having larger EAs than other commonly known systems, the search for strong electron acceptors is focused primarily on these compounds. Taking into account that the electronic stability of a monoanion strongly depends on the ability of excess charge delocalization over the molecular framework, one may anticipate that superhalogens containing large number of electronegative ligands should exhibit large excess electron binding energies. However, the number of ligands bound to a single central atom cannot be increased beyond certain values (mostly due to destabilizing valence repulsion effects and steric hindrance). Hence, polynuclear superhalogen anions matching the (M_n_X_n×k+1_)^–^ formula in which an excess electron density is expected to delocalize over n×*k*+1 electronegative ligands have been extensively studied in recent years (Alexandrova et al., 2004; Anusiewicz and Skurski, 2007; Freza and Skurski, 2010; Sikorska and Skurski, 2012; Wileńska et al., 2014; Yin et al., 2014; Li et al., 2015a, 2015b, Li et al., 2015 M.-M.; Czapla and Skurski, 2015, 2018; Díaz-Tinoco and Ortiz, 2016a; Díaz-Tinoco and Ortiz, 2016b; Sun et al., 2016b; Ding et al., 2017; Zhao et al., 2017; Anusiewicz et al., 2018; Cyraniak et al., 2019; Shi et al., 2019). Even though the polynuclear superhalogens investigated to date contain various central atoms (e.g., Li, Na, Mg, Ca, B, Al, Ge, Sn, P, Ti, Sb, As, V, In, Ta, Fe, Au, Pt), the systems utilizing nitrogen central atoms have not been proposed thus far. The lack of polynuclear superhalogens containing N central atoms seems intriguing and inspired us to make an attempt to propose and characterize such compounds. In addition, having in mind the well-known stability of saturated hydrocarbon structures, we decided to design our systems in a way that reflects the structures of chain-like C_n_H_2n+2_ molecules. In order to achieve that goal, we adopted the electronic transmutation concept which was introduced a decade ago.

Electronic transmutation is a concept introduced by Olson and Boldyrev (Olson and Boldyrev, 2012) who utilized the isoelectronic principle (Gillis, 1958) by proving that an element M with atomic number Z (i.e., _Z_M) is expected to undergo a transmutation into _Z+1_M *via* the acquisition of an extra electron. It was demonstrated (Alexandrova et al., 2003; Jemmis and Jayasree, 2003; Osorio et al., 2012; Popov and Boldyrev, 2013; Gish et al., 2015; Popov et al., 2015; Zhang et al., 2018a, 2018b; Lundell et al., 2020) that the resulting species (having Z+1 electrons) possesses the chemical bonding properties of the neighboring element _Z+1_M as if it was put in the place of the transmuted element _Z_M. Certainly, the same line of reasoning can be used for turning the element _Z_M into _Z–1_M by withdrawing one electron from it. Hence, we decided to design the structures containing the alternately aligned boron and nitrogen atoms (forming the (BN)_*n*_ ‘core’ of various length) and decorated with 4*n*+2 substituents (as if the B and N atoms comprising the core were carbon atoms). Indeed, assuming that each boron atom acquires an electron from its neighboring nitrogen atom, one may view the (BN)_*n*_ core as composed of alternately aligned B^−^and N^+^ ions, each of which is expected to mimic the bonding properties of a carbon atom (due to the presence of four valence electrons). As a result, the (BN)_*n*_ core might be expected to exhibit the bonding properties of the C_2*n*_ chain which naturally suggests the presence of 4*n*+2 substituents (to mimic the saturated hydrocarbon structure). Recalling that fluorine atoms are likely the most effective ligands in superhalogen systems, we decided to decorate the (BN)_*n*_ core with 4*n*+1 fluorine substituents and one BF_3_ substituent. The reason for using one BF_3_ substituent (instead of F) was that we wanted the whole system to represent a closed-shell superhalogen monoanion (BF_3_(BN)_*n*_F_4*n*+1_)^–^ rather than a closed shell neutral molecule (BN)_*n*_F_4*n*+2_.

Hence, in this contribution, we first describe the structures of the (BF_3_(BN)_*n*_F_4*n*+1_)^–^ (*n* = 1-10,13,18-20) systems followed by our theoretical findings concerning their thermodynamic stability and then we move on to discuss the vertical electron detachment energies these polynuclear superhalogen anions are characterized with.

## Methods

The stationary point structures of all systems investigated were obtained by applying the Density Functional Theory (DFT) method with the B3LYP (Becke, 1988; Lee et al., 1988) functional and the 6-311+G(d) (Krishnan et al., 1980; McLean and Chandler, 1980) basis set for all atoms. The harmonic vibrational frequencies characterizing the stationary points were evaluated (without scaling) at the same level of theory to assure that all obtained structures correspond to true minima on the potential energy surface. The vertical electron detachment energies of the (BF_3_(BN)_*n*_F_4*n*+1_)^–^ (*n* = 1-8) anions were calculated by applying the outer valence Green function OVGF method (*B* approximation) (Rowe, 1968; Simons, 1971; Cederbaum, 1975; Ortiz, 1988; Zakrzewski and Ortiz, 1994; Zakrzewski et al., 1996b) together with the 6-311+G(d) basis sets. Due to the limited computer resources available, the VDE values for larger systems (i.e., (BF_3_(BN)_*n*_F_4*n*+1_)^–^ (*n* = 9,10,13,18-20)) were estimated at the B3LYP/6-311+G(d) level of theory.

In order to verify the performance of the 6-311+G(d) basis set in predicting the VDE values of the anions studied we calculated (for (BF_3_(BN)F_5_)^–^ and (BF_3_(BN)_2_F_9_)^–^) the vertical electron detachment energies with two additional basis sets (i.e., aug-cc-pVTZ (Dunning, 1989) and Def2TZVP (Weigend and Ahlrichs, 2005)). As it turned out, the VDEs calculated at the OVGF/aug-cc-pVTZ level differ from those obtained by employing the OVGF/6-311+G(d) treatment by less than 0.2 eV (ca. 2%) whereas the VDEs calculated at the OVGF/Def2TZVP level differ by less than 0.3 eV (ca. 4%) from the OVGF/6-311+G(d) values. Since the above mentioned differences were found to be both relatively small and nearly insignificant (0.02–0.05 eV) for the larger anion tested (i.e., (BF_3_(BN)_2_F_9_)^–^), we conclude that our VDEs predicted with the 6-311+G(d) basis set can be considered reliable, especially for the (BF_3_(BN)_*n*_F_4*n*+1_)^–^, n > 1 anions).

As far as the performance of other DFT functionals in reproducing the preliminary estimates of the VDE values is concerned, we found (again, for (BF_3_(BN)F_5_)^–^ and (BF_3_(BN)_2_F_9_)^–^) that 1) employing the wB97XD functional (Chai and Head-Gordon, 2008) leads to the vertical electron detachment energies whose values are smaller by 0.08–0.12 eV than those predicted at the OVGF/6-311+G(d) level and larger by 0.20–0.49 eV than the values obtained with the B3LYP functional; 2) the use of the B2PLYPD functional (Grimme, 2006; Schwabe and Grimme, 2007) leads to the VDEs whose values are smaller by 0.22–0.36 eV than those predicted at the OVGF/6-311+G(d) level and larger by 0.10–0.14 eV than the values calculated with the B3LYP functional. Therefore, we conclude that our preliminary estimations of the VDEs characterizing the (BF_3_(BN)_*n*_F_4*n*+1_)^–^ anions are reliable yet the results obtained for two smallest systems considered indicate that the wB97XD functional performs best and thus it should be chosen if the VDE values were to be calculated only by DFT methods.

Due to the fact that the OVGF approximation remains valid only for outer valence ionization for which the pole strengths (PS) are greater than 0.80–0.85 (Zakrzewski et al., 1996a), we verified that the PS values obtained were sufficiently large to justify the use of the OVGF method.

The partial atomic charges were fitted to the electrostatic potential according to the Merz-Singh-Kollman scheme (Besler et al., 1990).

All calculations were carried out using the GAUSSIAN16 (Rev.B.01) package(Frisch et al., 2016).

## Results

In order to study the series of (BF_3_(BN)_*n*_F_4*n*+1_)^–^ anions, we decided to examine their structures for *n* = 1-10 and a few arbitrarily selected larger structures (for *n* = 13, 18-20). The reason was to verify whether the structures of larger (BF_3_(BN)_*n*_F_4*n*+1_)^–^ systems reflect those containing shorter (BN)_*n*_ core and to establish the approximate limit for the vertical electron detachment energy which could be achieved for these anions (presumably for large values of *n*).

### Equilibrium Structures and Stability of (BF_3_(BN)_*n*_F_4*n*+1_)^–^ (*n* = 1-10,13,18-20) Anions

**FIGURE 1:**
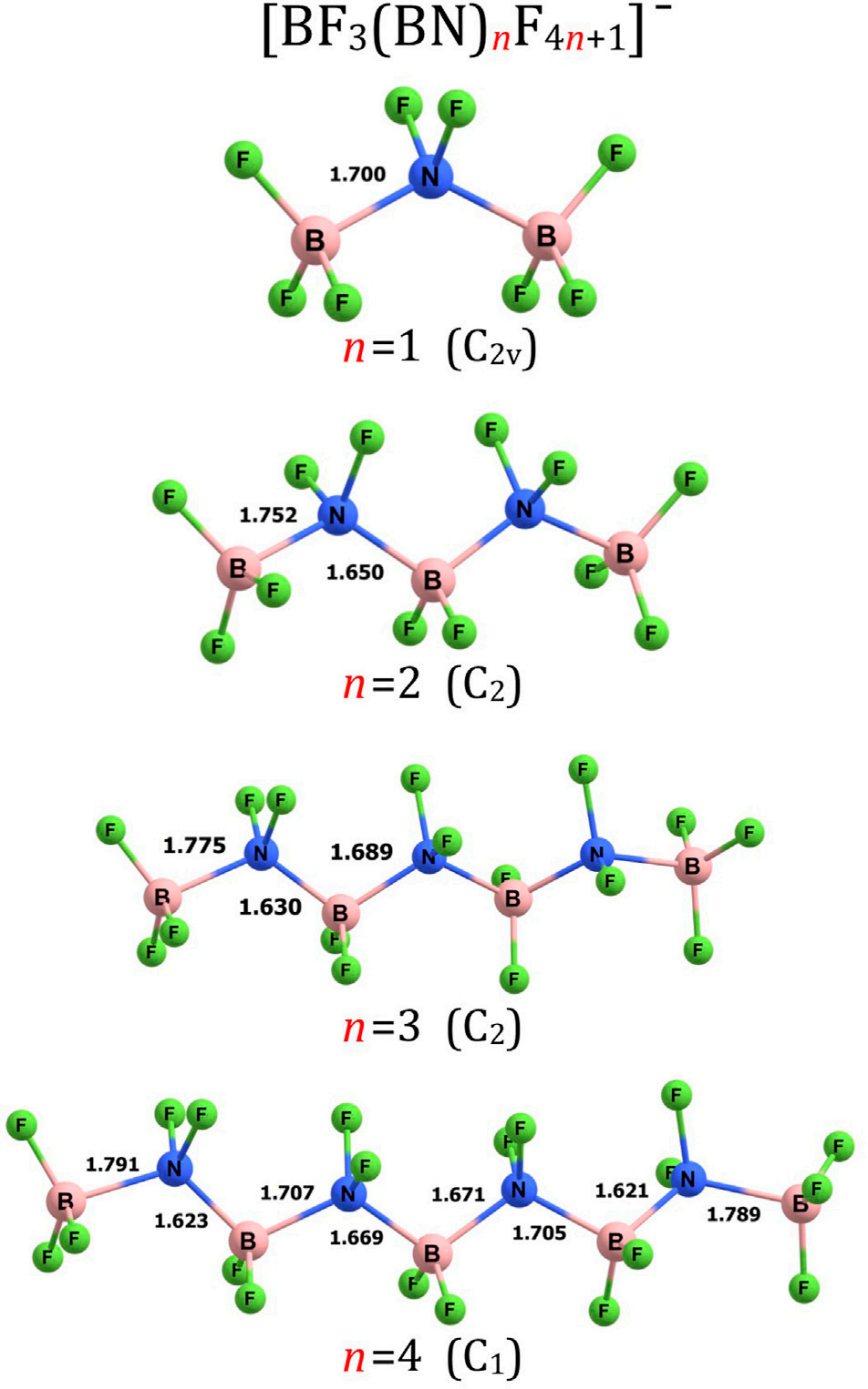
Equilibrium structures of (BF_3_(BN)_*n*_F_4*n*+1_)^–^ (*n* = 1–4) anions (symmetry point group in parenthesis). Selected bond lengths are given in Å.

The simplest anion matching the (BF_3_(BN)_*n*_F_4*n*+1_)^–^ formula corresponds to the (BF_3_BNF_5_)^–^ system (i.e., (BF_3_(BN)_*n*_F_4*n*+1_)^–^ for *n* = 1). We verified that its equilibrium structure is of C_2v_-symmetry and can be viewed as two BF_3_ groups connected to the central NF_2_ fragment (i.e., (BF_3_-NF_2_-BF_3_)^–^), see Figure 1. The length of both B-N bonds is equal to 1.700 Å, the length of both N-F bonds is equal to 1.399 Å, whereas the B-F bond lengths span the 1.376–1.382 Å range. As depicted in Figure 1, the substituents around each B and N central atom are arranged in a tetrahedral manner, as if B and N atoms were carbon atoms. In consequence, the structure of the (BF_3_-NF_2_-BF_3_)^–^ system resembles that of propane (or perfluoropropane) which in turn indicates that one may consider the electronic transmutation of B and N atoms in (BF_3_-NF_2_-BF_3_)^–^ accomplished. We verified that the (BF_3_-NF_2_-BF_3_)^–^ is actually the only geometrically stable isomer of the (BF_3_BNF_5_)^–^ system as all attempts to find the structures having different arrangement of atoms failed (i.e., the geometry optimizations of various initial structures resulted in fragmentation of the system). As far as the thermodynamic stability of (BF_3_-NF_2_-BF_3_)^–^ is concerned, we considered several fragmentation paths leading to various molecular fragments (such as BF_4_^−^, F_2_^−^, F_2_, NF_3_, BF_3_, NF_4_^−^, etc.) and we verified that none of those paths is energetically favorable. Since we have also proven that the (BF_3_-NF_2_-BF_3_)^–^ anion is electronically stable (by verifying that its excess electron binding energy is positive, see the following section for details), we are confident that the (BF_3_BNF_5_)^–^ system is a thermodynamically stable species.

The structure of (BF_3_(BN)_*n*_F_4*n*+1_)^–^ for *n* = 2 exhibits C_2_-symmetry and contains alternately aligned B and N central atoms forming the B-N-B-N-B chain decorated with 12 fluorine ligands, see Figure 1. As it was the case for the (BF_3_-NF_2_-BF_3_)^–^ anion, we found only one geometrically stable structure of (BF_3_-NF_2_-BF_2_-NF_2_-BF_3_)^–^ despite the fact that we considered a large number of alternative structures having various arrangements of B, N and F atoms, including not only chain-like structures but also branched systems. Nevertheless, all these alternative initial structures turned out to be geometrically unstable. The terminal B-N bonds in (BF_3_-NF_2_-BF_2_-NF_2_-BF_3_)^–^ were found to be longer (by 0.052 Å) than the B-N bonds in (BF_3_-NF_2_-BF_3_)^–^ whereas the remaining B-N bonds in the former system were predicted to be shorter (by 0.050 Å) than those in the latter one. The B-F and N-F bond lengths in (BF_3_-NF_2_-BF_2_-NF_2_-BF_3_)^–^ were found to be slightly shorter than the corresponding separations in (BF_3_-NF_2_-BF_3_)^–^ as they span the 1.352–1.371 and 1.390–1.394 Å range, respectively. Tetrahedral arrangement of the substituents around each B and N atom causes the (BF_3_-NF_2_-BF_2_-NF_2_-BF_3_)^–^ structure to resemble that of n-pentane (as if all boron and nitrogen atoms were mimicking the bonding pattern typical for carbon atoms).

**FIGURE 2:**
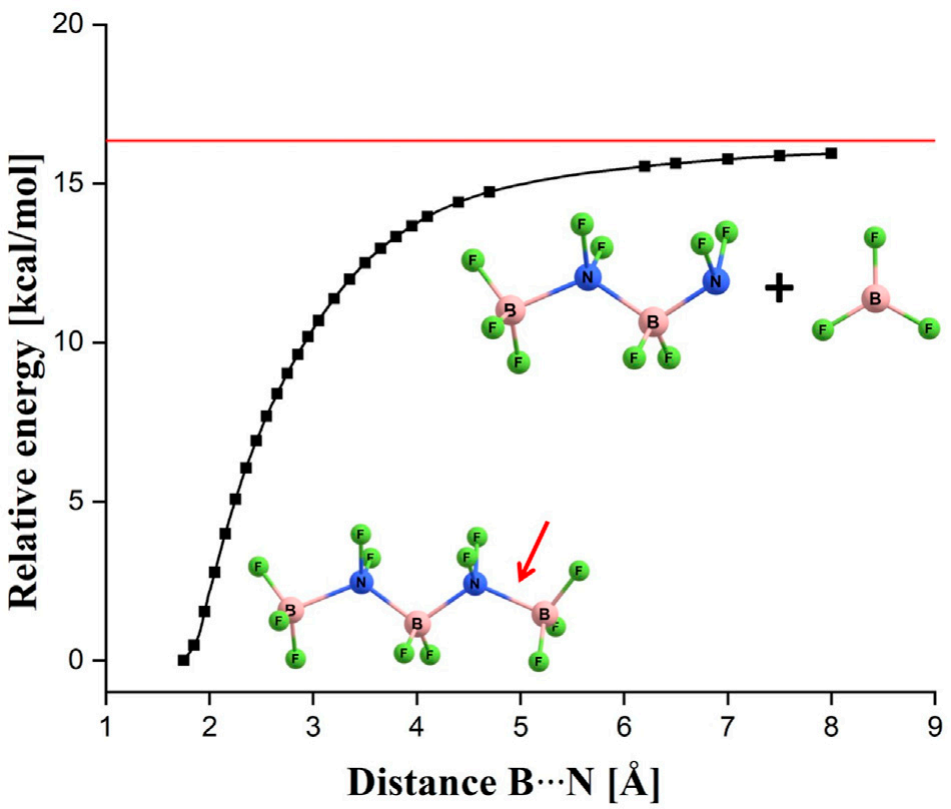
Energy profile corresponding to the relaxed scan along the terminal B-N bond (indicated by a red arrow) in the (BF_3_-NF_2_-BF_2_-NF_2_-BF_3_)^–^ anion. The horizontal red line indicates the sum of the energies of the isolated (BF_3_-NF_2_-BF_2_-NF_2_)^–^ and BF_3_ systems.

Since the (BF_3_-NF_2_-BF_2_-NF_2_-BF_3_)^–^ system (i.e., BF_3_(BN)_*n*_F_4*n*+1_)^–^ for *n* = 2) is larger than (BF_3_-NF_2_-BF_3_)^–^ anion (i.e., BF_3_(BN)_*n*_F_4*n*+1_)^–^ for *n* = 1) (and thus it is likely more similar to larger (BF_3_(BN)_*n*_F_4*n*+1_)^–^ (*n* > 2) systems than the latter) yet small enough to enable a comprehensive analysis of its possible fragmentation channels, we decided to choose this particular species to verify whether the more complex (BF_3_(BN)_*n*_F_4*n*+1_)^–^ anions might be vulnerable to fragmentation processes. Hence, we considered the possible loss of various molecular fragments from (BF_3_(BN)_2_F_9_)^–^ by calculating the energies of various fragmentation products and then comparing them to the energy of (BF_3_(BN)_2_F_9_)^–^. We found one (and only) case in which the fragments are lower in energy than the (BF_3_-NF_2_-BF_2_-NF_2_-BF_3_)^–^ anion, namely, we verified that the energy sum of BF_3_ and a branched (BF_3_-NF(BF_3_)-NF_2_)^–^ system is smaller by about 24 kcal/mol than the energy of (BF_3_-NF_2_-BF_2_-NF_2_-BF_3_)^–^. From the formal point of view, this finding indicates thermodynamic instability of (BF_3_(BN)_2_F_9_)^–^, however, one should also consider the fragmentation path which could potentially be pursued to generate such a set of species. Obviously, the process of transformation of the (BF_3_-NF_2_-BF_2_-NF_2_-BF_3_)^–^ anion into BF_3_ and (BF_3_-NF(BF_3_)-NF_2_)^–^ fragments would have to proceed according to some stepwise mechanism involving the detachment of BF_3_ molecule from (BF_3_-NF_2_-BF_2_-NF_2_-BF_3_)^–^ followed by the substantial reorganization of the remaining (BF_3_-NF_2_-BF_2_-NF_2_)^–^ anion (in order to produce a final branched (BF_3_-NF(BF_3_)-NF_2_)^–^ structure). Having this in mind, we performed a relaxed scan of the potential energy surface of (BF_3_-NF_2_-BF_2_-NF_2_-BF_3_)^–^ along the terminal N-B bond, see Figure 2. Our calculations revealed that a spontaneous detachment of the BF_3_ molecule from the (BF_3_-NF_2_-BF_2_-NF_2_-BF_3_)^–^ anion would be energetically unfavorable by ca. 16 kcal/mol (as the energy profile shown in Figure 2 affirms) and thus should be considered not likely. Therefore, we conclude that the (BF_3_(BN)_2_F_9_)^–^ isomer, although higher in energy than the (BF_3_-NF(BF_3_)-NF_2_)^–^ + BF_3_ fragments, should remain stable. Moreover, we believe that we can extend that conclusion to cover also larger (BF_3_(BN)_*n*_F_4*n*+1_)^–^ (*n* > 2) systems by assuming that our considerations based on the *n* = 2 case can be treated as representative for longer chain-like (BF_3_(BN)_*n*_F_4*n*+1_)^–^ anions whose structures we are about to discuss. In other words, we assume that all larger (BF_3_(BN)_*n*_F_4*n*+1_)^–^ (*n* > 2) systems we consider in this work are likely not thermodynamically stable with respect to the formation of BF_3_ and the remaining branched anionic fragment, yet should be long lived due to the fact that the BF_3_ loss which would have to happen to trigger such a process is energetically unfavorable.

**FIGURE 3:**
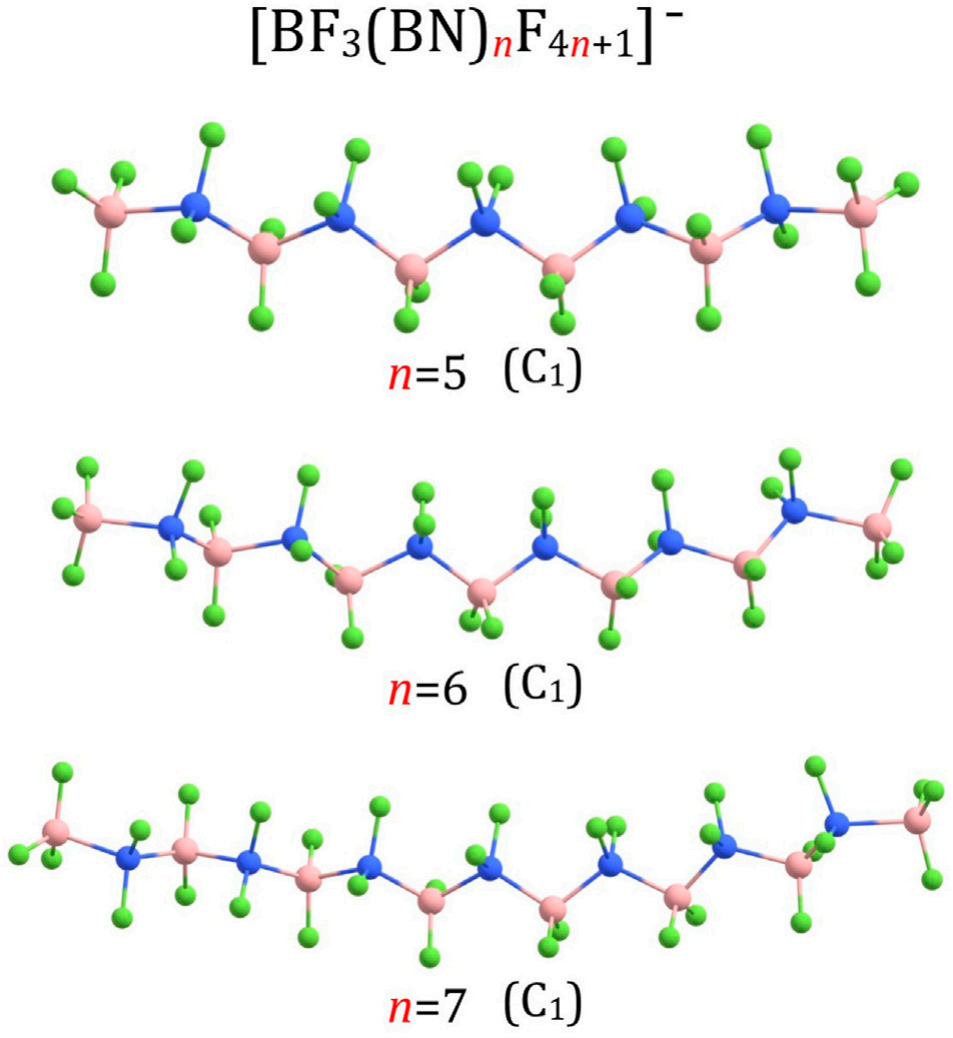
Equilibrium structures of (BF_3_(BN)_*n*_F_4*n*+1_)^–^ (*n* = 5–7) anions (symmetry point group in parenthesis).

**FIGURE 4:**
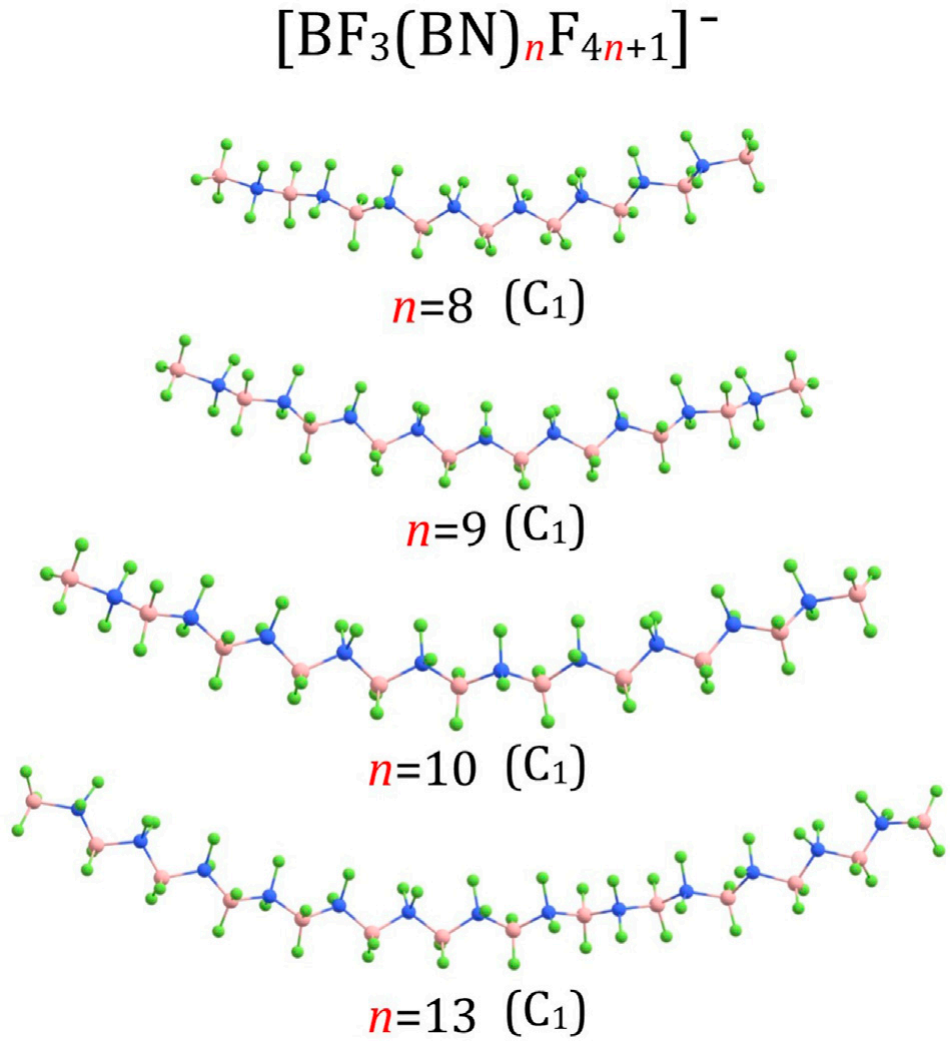
Equilibrium structures of (BF_3_(BN)_*n*_F_4*n*+1_)^–^ (*n* = 8–10,13) anions (symmetry point group in parenthesis).

**FIGURE 5:**
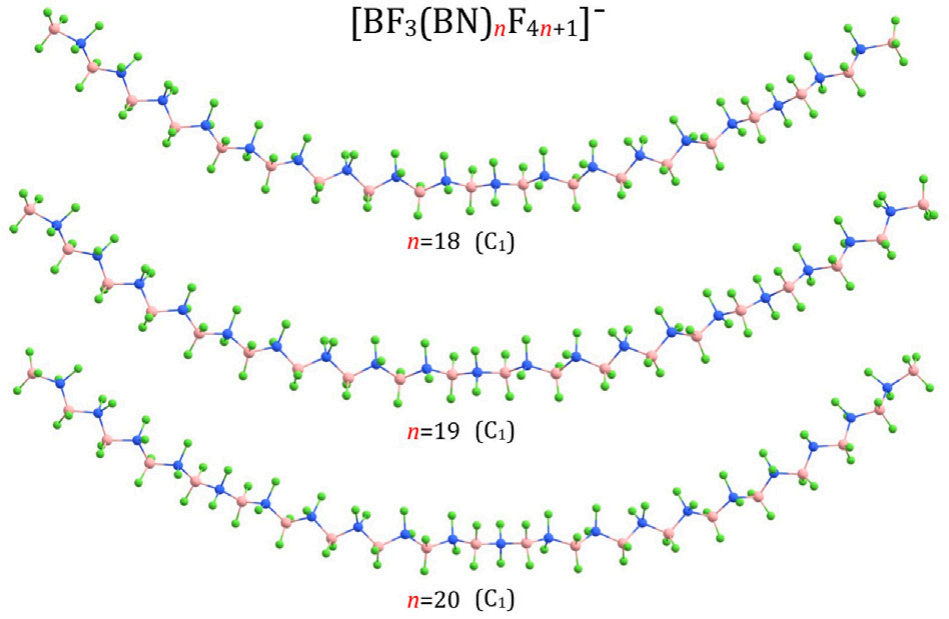
Equilibrium structures of (BF_3_(BN)_*n*_F_4*n*+1_)^–^ (*n* = 18–20) anions (symmetry point group in parenthesis).

Since the structures of larger (BF_3_(BN)_*n*_F_4*n*+1_)^–^ anions (i.e., for *n* = 3-10, 13, 18-20) resemble many similarities with one another, we describe them together in this section. The (BF_3_(BN)_*n*_F_4*n*+1_)^–^ structures for *n* = 3 and *n* = 4 are shown in Figure 1, the structures for *n* = 5-7 are presented in Figure 3, the structures for *n* = 8-10 and *n* = 13 are depicted in Figure 4, whereas the structures for *n* = 18-20 are gathered in Figure 5.

Our calculations revealed similarities among the corresponding B-N, B-F, and N-F bond lengths in the (BF_3_(BN)_*n*_F_4*n*+1_)^–^ anions considered (i.e., for *n* = 1-10, 13, 18-20). Namely, the B-N bonds in (BF_3_(BN)_3_F_13_)^–^ (1.630–1.775 Å) and (BF_3_(BN)_4_F_17_)^–^ (1.621–1.791 Å) are of similar lengths to those found for (BF_3_(BN)F_5_)^–^ and (BF_3_(BN)_2_F_9_)^–^, see the discussion in the preceding paragraphs. In addition, B-N bond lengths predicted for larger systems (1.616–1.797 Å for *n* = 5, 1.613–1.805 Å for *n* = 6, 1.611–1.807 Å for *n* = 7, 1.610–1.811 Å for *n* = 8, 1.609–1.812 Å for *n* = 9, 1.608–1.813 Å for *n* = 10, 1.607–1.815 Å for *n* = 13, and 1.607–1.817 Å for *n* = 18-20) show an analogous pattern (i.e., slightly larger separations between terminal BF_3_ groups and the remaining molecular fragment). The N-F bonds in the (BF_3_(BN)_*n*_F_4*n*+1_)^–^ (*n* > 2) anions (1.387–1.394 Å for *n* = 3, 1.383–1.394 Å for *n* = 4, 1.384–1.394 Å for *n* = 5, 1.383–1.394 Å for *n* = 6, 1.381–1.394 Å for *n* = 7, 1.380–1.394 Å for *n* = 8-10 and *n* = 13, 1.379–1.394 Å for *n* = 18-20) are also similar to those in (BF_3_(BN)_3_F_13_)^–^ and (BF_3_(BN)_4_F_17_)^–^. The B-F bond lengths in the (BF_3_(BN)_*n*_F_4*n*+1_)^–^ (*n* > 2) systems span the following ranges: 1.347–1.366 Å for *n* = 3, 1.342–1.365 Å for *n* = 4 and *n* = 5, 1.338–1.363 Å for *n* = 6-9, 1.336–1.362 Å for *n* = 10, *n* = 13, and *n* = 18-20. In addition to the similar lengths of the corresponding B-N, B-F, and N-F bonds, the substituents around each B and N atom in the (BF_3_(BN)_*n*_F_4*n*+1_)^–^ (*n* = 3-10, 13, 18-20) anions are arranged in a tetrahedral manner as it was the case for (BF_3_(BN)_3_F_13_)^–^ and (BF_3_(BN)_4_F_17_)^–^, see Figure 1 and Figures 3–5.

According to our predictions, the structures of (BF_3_(BN)_*n*_F_4*n*+1_)^–^ anions correspond to extended conformations with a tendency to form arched chains when the number of BN units (*n*) develops. In general, the structures predicted for (BF_3_(BN)_*n*_F_4*n*+1_)^–^ systems resemble the extended conformations of higher n-alkanes. Indeed, as it was established in the earlier studies (Lüttschwager et al., 2013), linear alkanes of moderate length (i.e., containing up to 17(Thomas et al., 2006) or 21 (Grimme et al., 2007) carbon atoms (depending on the research method used)) tend to adopt a fully extended (all-*trans*) conformations at low temperatures, whereas weak dispersion interactions between chain fragments come into play for larger systems by causing the *trans-gauche* isomerizations which eventually transform an extended chain into a hairpin structure (Lüttschwager et al., 2013). Therefore, we believe that the structures of the (BF_3_(BN)_*n*_F_4*n*+1_)^–^ (*n* = 1-10) anions presented in Figures 1, 3, 4 likely correspond to globally stable conformations as the number of B and N atoms (each of which mimics a carbon atom due to electronic transmutation) does not exceed 21, hence it approaches the maximum number of C atoms for which the fully extended alkanes remain to be globally stable structures. As far as the (BF_3_(BN)_*n*_F_4*n*+1_)^–^ (*n* = 13,18-20) anions are concerned, the possible existence of alternative lower energy conformers should be considered likely.

### Excess Electron Binding Energies of (BF_3_(BN)_*n*_F_4*n*+1_)^–^ (*n* = 1-10,13,18-20) Anions

The population analysis performed according to the Merz-Singh-Kollman scheme revealed that the excess negative charge in all (BF_3_(BN)_*n*_F_4*n*+1_)^–^ (*n* = 1-10,13,18-20) anions is distributed among the fluorine ligands, yet not evenly. In order to simplify the discussion, we decided to describe the partial atomic charges obtained for two structurally smallest systems, (BF_3_(BN)F_5_)^–^ and (BF_3_(BN)_2_F_9_)^–^ which can be considered representative for all (BF_3_(BN)_*n*_F_4*n*+1_)^–^ anions studied in this work. In the case of (BF_3_(BN)F_5_)^–^ system (i.e., (BF_3_-NF_2_-BF_3_)^–^), the partial charges (*q*) localized on the F atoms connected to boron atoms are equal to –0.43|e| whereas those localized on the F atoms linked to the nitrogen atom are equal to –0.11|e| (naturally, all partial charges sum up to –1|e| as the partial charges predicted for two boron atoms and for one nitrogen atom are equal to +0.92|e| and –0.02|e|, respectively). In the case of (BF_3_(BN)_2_F_9_)^–^ anion (i.e., (BF_3_-NF_2_-BF_2_-NF_2_-BF_3_)^–^), the *q* ^F^ charges determined for the F atoms connected to the terminal B atoms are equal to ca. –0.41|e|, the *q* ^F^ of –0.32|e| are predicted for fluorine atoms bound to the central B atom, whereas *q* ^F^ for the F atoms linked to N atoms are equal to –0.09|e|. Since partial atomic charges determined for larger (BF_3_(BN)_*n*_F_4*n*+1_)^–^ (*n* > 2) anions exhibit approximately the same pattern as we found for these two smallest systems (for *n* = 1,2), one may arrive at the following generalizations: 1) the excess negative charge is delocalized among all fluorine ligands (which is reasonable taking into account the substantial electronegativity of F atoms), 2) the atomic partial charges localized on the F, N, and B atoms constituting the central part of a chain (i.e., the chain without two terminal BF_3_ groups) sum up to approximately zero, and 3) the atomic partial charges on B and F atoms comprising each terminal BF_3_ group sum up to ca. −0.5|e|. Therefore, we conclude that the excess negative charge in the anions considered is localized mainly on the two terminal BF_3_ fragments.

**FIGURE 6:**
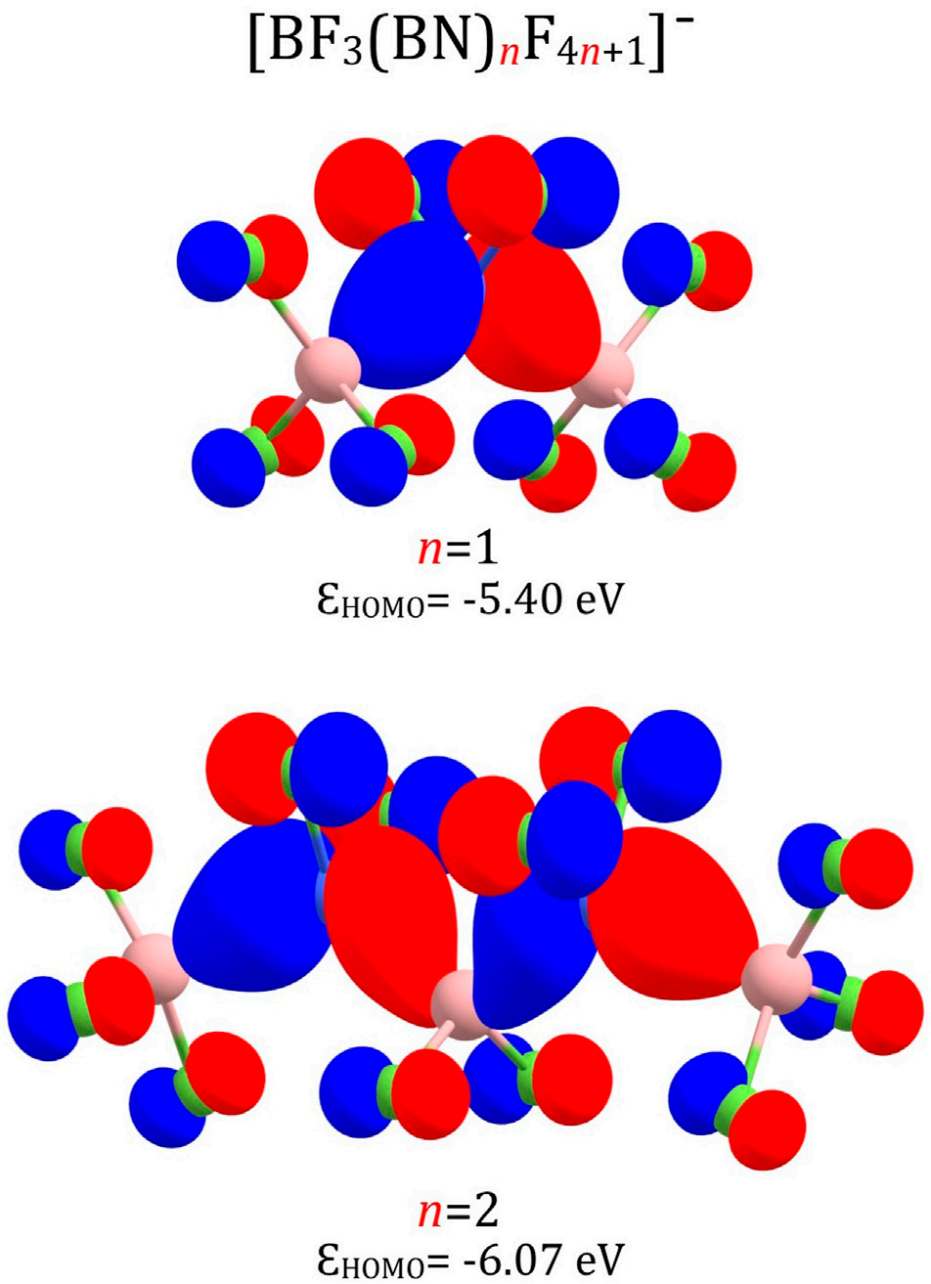
HOMO orbitals of the (BF_3_(BN)F_5_)^–^ and (BF_3_(BN)_2_F_9_)^–^ anions with their orbital energies (*ε*).

The highest occupied molecular orbitals (HOMO) of the smallest (BF_3_(BN)_*n*_F_4*n*+1_)^–^ anions (i.e., (BF_3_(BN)F_5_)^–^ and (BF_3_(BN)_2_F_9_)^–^) exhibit the bonding pattern typical for almost all superhalogen anions described in the literature(Skurski, 2021), see Figure 6. Indeed, one may easily notice the absence of destabilizing antibonding ligand-central atom interactions, which is characteristic not only for (MX_k+1_)^–^ systems containing one central atom but also for polynuclear (M_n_X_n×k+1_)^–^ superhalogen anions. In addition, the analysis of HOMO for (BF_3_(BN)F_5_)^–^ and (BF_3_(BN)_2_F_9_)^–^ reveals strong bonding boron-nitrogen interactions and substantial contributions from 2*p* atomic orbitals of fluorine atoms. Since the HOMOs calculated for larger (BF_3_(BN)_*n*_F_4*n*+1_)^–^ anions (*n* > 2) look alike, we do not present them here (as the HOMO contour plots for *n* = 1 and *n* = 2 are representative for all systems considered).

The vertical electron detachment energies predicted for the (BF_3_(BN)_*n*_F_4*n*+1_)^–^ anions (*n* = 1-10,13,18-20) are collected in Table 1. In fact, we present the VDEs calculated by using the OVGF method (for *n* = 1-8) and the B3LYP method (for *n* = 1-10,13,18-20). Certainly, the VDE values obtained by employing the OVGF method (VDE^OVGF^) are much more reliable than those determined by the use of the B3LYP method (VDE^B3LYP^), however, our computer resources enabled performing the calculations of VDE^OVGF^ values only for the (BF_3_(BN)_*n*_F_4*n*+1_)^–^ anions up to *n* = 8. Therefore, we decided to determine the VDEs for the larger systems (i.e., for *n* = 9,10,13,18-20) by employing a less computationally demanding B3LYP approach. In fact, we applied the B3LYP method to predict the VDEs also for those systems (*n* = 1-8) whose vertical electron detachment energies were calculated with the OVGF method (to enable the comparison between the OVGF and B3LYP results which allowed us to assess the reliability of the VDE^B3LYP^ values). Hence, we discuss the VDEs of the (BF_3_(BN)_*n*_F_4*n*+1_)^–^ anions based on the VDE^OVGF^ values whereas the VDE^B3LYP^ results we use only to predict a likely VDE dependence on *n* (i.e., the VDE = *f* (*n*) function) and thus to estimate the approximate values of VDE^OVGF^ for *n* = 9,10,13,18-20.

**TABLE 1:** Vertical electron detachment energies (in eV) of the (BF_3_(BN)_*n*_F_4*n*+1_)^–^ anions (*n* = 1-10,13,18-20) determined at the OVGF/6-311+G(d) (labeled VDE^OVGF^) and B3LYP/6-311+G(d) (labeled VDE^B3LYP^) level of theory. ΔVDE stands for the difference between VDE^OVGF^ and VDE^B3LYP^.

System	VDE^OVGF^ (eV)	VDE^B3LYP^ (eV)	ΔVDE (eV)
(BF_3_(BN)F_5_)^–^	8.10	7.78	0.32
(BF_3_(BN)_2_F_9_) ^–^	8.57	8.07	0.50
(BF_3_(BN)_3_F_13_)^–^	9.07	8.35	0.72
(BF_3_(BN)_4_F_17_)^–^	9.45	8.53	0.92
(BF_3_(BN)_5_F_21_)^–^	9.77	8.68	1.09
(BF_3_(BN)_6_F_25_)^–^	9.96	8.76	1.20
(BF_3_(BN)_7_F_29_)^–^	10.10	8.84	1.26
(BF_3_(BN)_8_F_33_)^–^	10.18	8.89	1.29
(BF_3_(BN)_9_F_37_)^–^	∼10.33^a^	8.92	1.41
(BF_3_(BN)_10_F_41_)^–^	∼10.41^a^	8.95	1.46
(BF_3_(BN)_13_F_53_)^–^	∼10.55^a^	9.01	1.54
(BF_3_(BN)_18_F_73_)^–^	∼10.64^a^	9.05	1.59
(BF_3_(BN)_19_F_77_)^–^	∼10.65^a^	9.06	1.59
(BF_3_(BN)_20_F_81_)^–^	∼10.66^a^	9.07	1.59

a alues extrapolated on the basis of the exponential fitting function, see caption for Figure 7.

We start our discussion with recalling the fact that the VDE of the (BF_4_)^–^ was earlier calculated to be 8.98 eV (Sikorska et al., 2008). We consider this result important because the (BF_4_)^–^ system matches the (BF_3_(BN)_*n*_F_4*n*+1_)^–^ formula for *n* = 0. The VDE of the smallest anion investigated in this work ((BF_3_(BN)F_5_)^–^, which can be treated as the (BF_4_)^–^ system having one of its ligands replaced with the NF_2_-BF_3_ fragment) was evaluated as equal to 8.10 eV, see Table 1. We believe the reason why the VDE of (BF_3_(BN)F_5_)^–^ is smaller than that of (BF_4_)^–^ is that replacing one F ligand with NF_2_-BF_3_ fragment lowers the symmetry of the system which in turn causes the decrease of the excess electron binding energy (as it was established for various superhalogen anions (Smuczyńska and Skurski, 2008)). However, the molecular fragment (BN)_*n*_F_4*n*+1_ that the F atom is replaced with contains more and more electronegative fluorine ligands when *n* develops and thus the VDE of the (BF_3_(BN)_*n*_F_4*n*+1_)^–^ anion increases when *n* increases, see Table 1. In particular, the VDE of 8.57 eV was calculated for *n* = 2 and the VDE of 9.07 eV was predicted for *n* = 3. As one can notice, the VDE found for (BF_3_(BN)_3_F_13_)^–^ slightly exceeds that found for the reference (BF_4_)^–^ anion, which means that the presence of electronegative ligands in the (BN)_3_F_13_ fragment compensates (in terms of the excess electron binding energy) the destabilizing effects related to the symmetry lowering.

The VDE predicted for larger (BF_3_(BN)_*n*_F_4*n*+1_)^–^ anions gradually increases to achieve the value of 10.18 eV for *n* = 8, see Table 1. Although one might anticipate the continuation of this tendency for larger *n* values, we cannot provide the precise numeric values due to the lack of the OVGF-based results for *n* > 8. Despite this, we made an attempt to estimate the VDEs of the larger systems considered (i.e., (BF_3_(BN)_*n*_F_4*n*+1_)^–^ for *n* > 8) by finding the approximate function VDE^OVGF^ = *f* (*n*) and extrapolating it to achieve the VDE^OVGF^ for *n* = 20. In order to do this, we decided to choose the fitting function given by the ${\text{VDE}}^{\text{OVGF}}=A\cdot exp\left(-n/B\right)+C$ formula (where *A*, *B*, and *C* are the fitting parameters) because we verified that such a function properly describes the set of VDE^B3LYP^ values we obtained for all (BF_3_(BN)_*n*_F_4*n*+1_)^–^ anions (*n* = 1-10,13,18-20) which the VDE^B3LYP^ = *f* (*n*) plot depicted in Figure 7 affirms. In other words, we assume that the VDE^B3LYP^ values, although clearly underestimated with respect to more reliable VDE^OVGF^ values (see Table 1), show the proper VDE trend for developing *n*. According to the fitting function obtained for the VDE^OVGF^ results, the vertical electron detachment energy of the (BF_3_(BN)_20_F_81_)^–^ anion is approximately equal to 10.7 eV. Although this value comes from the extrapolation to *n* = 20, we consider this estimate rather reliable because the *r* ^2^ (i.e., coefficient of determination) for that fit approaches 1.0 (0.99665, see the caption for Figure 7). Both the shape of the VDE^OVGF^ = *f* (*n*) plot and the fact that the differences between the consecutive VDE^OVGF^ values for *n* developing from 6 to 8 are small (ca. 0.1 eV) indicate that the extrapolated VDE of 10.7 eV for *n* = 20 may actually approach the maximal VDE which can be obtained for (BF_3_(BN)_*n*_F_4*n*+1_)^–^ polynuclear superhalogen anions (even for *n* > 20).

**FIGURE 7:**
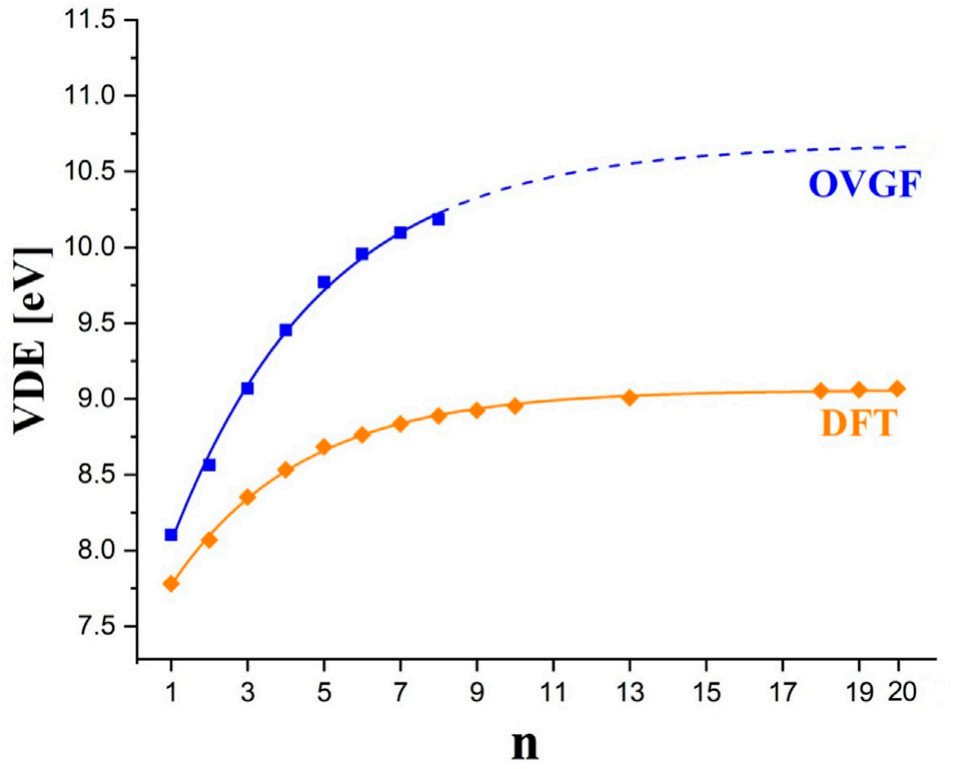
The VDE^OVGF^ values (blue squares) and VDE^B3LYP^ values (orange diamonds) calculated for (BF_3_(BN)_*n*_F_4*n*+1_)^–^ anions. The plots correspond to the fitting formula given by VDE=A⋅exp(−n/B)+C (where *A*, *B*, and *C* stand for fitting parameters). The fitted parameters *A* = −3.36783 ± 0.09853, *B* = 4.00503 ± 0.45838, and *C* = 10.68291 ± 0.14242 and the coefficient of determination *r* ^2^ = 0.99665 were obtained for the function approximating VDE^OVGF^ results (blue line) while *A* = −1.72646 ± 0.02308, *B* = 3.40804 ± 0.08137, and *C* = 9.03863 ± 0.00786 and the *r* ^2^ of 0.99878 were obtained for that approximating VDE^B3LYP^ results (orange line).

## Conclusion

On the basis of the B3LYP/6-311+G(d) and OVGF/6-311+G(d) calculations (whose accuracy were verified by employing the wB97XD/6-311+G(d), B2PLYPD/6-311+G(d), OVGF/aug-cc-VTZ, and OVGF/Def2TZVP treatments) performed for the (BF_3_(BN)_*n*_F_4*n*+1_)^–^ (*n* = 1-10,13,18-20) anions we arrive at the following conclusions:

1) The electronic transmutation concept can be employed to design polynuclear superhalogen anions matching the (BF_3_(BN)_*n*_F_4*n*+1_)^–^ formula and comprising alternately aligned boron and nitrogen central atoms.
2) The equilibrium structures of (BF_3_(BN)_*n*_F_4*n*+1_)^–^ (*n* = 1-10,13,18-20) anions correspond to fully extended (all-*trans*) chains with four substituents arranged in a tetrahedral manner around each B and N central atom and thus mimic the globally stable fully extended conformations of higher n-alkanes.
3) The excess negative charge in (BF_3_(BN)_*n*_F_4*n*+1_)^–^ (*n* = 1-10,13,18-20) anions is delocalized mainly among the fluorine ligands attached to two terminal boron atoms.
4) The vertical electron detachment energies predicted for (BF_3_(BN)_*n*_F_4*n*+1_)^–^ (*n* = 1-8) anions always exceed 8 eV, gradually increase with developing *n* and approach 10.2 eV for *n* = 8.
5) The estimated VDE value for *n* = 20 (i.e., for the (BF_3_(BN)_20_F_81_)^–^ system) is about 10.7 eV and it is anticipated to represent the upper limit of vertical electron binding energy which could be achieved for polynuclear superhalogen anions matching the (BF_3_(BN)_*n*_F_4*n*+1_)^–^ formula.

## Data Availability

The original contributions presented in the study are included in the article/Supplementary Material, further inquiries can be directed to the corresponding author.
